# Investigating the mediating effect of working memory on intentional forgetting in dysphoria

**DOI:** 10.1007/s00426-019-01225-y

**Published:** 2019-07-19

**Authors:** Saima Noreen, Richard Cooke, Nathan Ridout

**Affiliations:** 1 School of Allied Health Sciences, Faculty of Life and Health Sciences, De Montfort University, Leicester, UK; 2 Institute of Psychology Health and Society, University of Liverpool, Liverpool, UK; 3 Department of Psychology, School of Life and Health Sciences, Aston University, Birmingham, B4 7ET UK

## Abstract

Our aim was to determine if deficits in intentional forgetting that are associated with depression and dysphoria (subclinical depression) could be explained, at least in part, by variations in working memory function. Sixty dysphoric and 61 non-dysphoric participants completed a modified version of the think/no-think (TNT) task and a measure of complex working memory (the operation span task). The TNT task involved participants learning a series of emotional cue–target word pairs, before being presented with a subset of the cues and asked to either recall the associated target (think) or to prevent it from coming to mind (no think) by thinking about a substitute target word. Participants were subsequently asked to recall the targets to all cues (regardless of previous recall instructions). As expected, after controlling for anxiety, we found that dysphoric individuals exhibited impaired forgetting relative to the non-dysphoric participants. Also as expected, we found that superior working memory function was associated with more successful forgetting. Critically, in the dysphoric group, we found that working memory mediated the effect of depression on intentional forgetting. That is, depression influenced forgetting indirectly via its effect on working memory. However, under conditions of repeated suppression, there was also a direct effect of depression on forgetting. These findings represent an important development in the understanding of impaired forgetting in depression and also suggest that working memory training might be a viable intervention for improving the ability of depressed individuals to prevent unwanted memories from coming to mind.

## Introduction

It has been well established that negative thoughts and biased cognition are central to the development and the maintenance of depression (Beck & Alford, 2009; Beck & Clark, 1988; Bellew & Hill, 1990; Hamilton & Gotlib, 2008; Joormann, Hertel, Brozovich & Gotlib, 2005). Critically, depressed individuals often fail to prevent irrelevant material from coming to mind (Gotlib & Joormann, 2010; Joormann & Gotlib, 2008). For example, there is a growing body of work showing that individuals with clinical and subclinical depression (referred to as dysphoria) have difficulties in preventing unwanted memories from coming to mind (Hertel & Gerstle, 2003; Howell & Conway, 1992; Joormann, Hertel, LeMoult & Gotlib, 2009; Joormann & Tran, 2009; Noreen & Ridout, 2016a, b). Interestingly, evidence suggests that attempts by depressed participants to suppress unwanted memories often lead to enhanced memory for this ‘to-be-forgotten’ material (e.g. Noreen & Ridout, 2016a, b).

Intentional forgetting has often been studied using the Think/No-Think paradigm (TNT; Anderson & Green, 2001). In this task, participants learn a series of (cue–target) word pairs before being presented with the cues from a subset of these pairs and asked to recall the associated target word to some cues (think condition) and to avoid recalling the target word to other cues (no-think condition). It has consistently been found in healthy participants that ‘not thinking’ about the associated targets leads to forgetting of these words on subsequent memory tests, which is referred to as ‘suppression-induced forgetting’ (Depue, Curran & Banich, 2007; Noreen & MacLeod, 2013, 2014; Noreen, Bierman & MacLeod, 2014; Anderson & Huddleston, 2011). On the other hand, depressed individuals typically exhibit difficulties in their ability to suppress their memory for the target words. For example, Joormann et al. (2009) used the TNT task and found that clinically depressed participants showed impaired forgetting of negative words. Impaired forgetting on the TNT has also been observed in participants with dysphoria (Hertel & Gerstle, 2003; Noreen & Ridout, 2016a, b), although the deficit in these participants was not limited to negative words.

There is a large body of research which suggests that suppression-induced forgetting effects on the TNT are due to an inhibitory mechanism that disrupts the availability of the unwanted memory, which later renders it inaccessible (Anderson & Hansmayr, 2014; Anderson et al., 2004). With this in mind, it is notable that depression is associated with deficits in inhibitory control (Joormann, Yoon & Zetsche, 2007; Joormann, 2004; Goeleven, De Raedt, Baert, & Baert, 2006). Therefore, it has been suggested that impaired forgetting in depression is likely to be a consequence of poor inhibitory control (Noreen & Ridout, 2016a). Supporting evidence for this comes from findings (e.g. Davidson, Pizzagalli, Nitschke, & Putnam, 2002) that depression is associated with reduced activity in the anterior cingulate cortex (ACC) and the dorsolateral prefrontal cortex (DLPFC), as these regions are activated during memory suppression (Anderson et al., 2004; Depue et al., 2007; Benoit & Anderson, 2012; Gagnepain, Henson & Anderson, 2014; Benoit, Hulbert, Huddleston, & Anderson, 2015; Depue, Orr, Smolker, Naaz, & Banich, 2015). Further evidence comes from Zhang, Xie, Liu and Luo (2016) who measured EEG activity and reported that suppression-induced forgetting of negative material in depressed individuals was associated with reduced frontal N2 activity, which the authors suggested reflected reduced voluntary inhibition of the words.

Another factor that has been implicated in intentional forgetting is working memory capacity. For example, Aslan and Bäuml (2011) used the retrieval practice paradigm, which measures adaptive forgetting with the retrieval of relevant information decreasing the access of related information (known as retrieval-induced forgetting) and found that working memory capacity, as measured by the operation span task (OSPAN, Turner & Engle, 1989), was positively correlated with retrieval-induced forgetting. More recently, Noreen and De Fockert (2017) conducted two studies that involved participants completing the TNT task whilst simultaneously performing a modified version of the n-back task and found that participants demonstrated lower levels of suppression-induced forgetting under high, compared to low, working memory load. These findings are consistent with studies demonstrating that individuals with good working capacity are more successful at inhibiting distracting information. For example, Brewin & Beaton (2002) used the standard ‘white bear’ paradigm and the OSPAN and reported that greater working memory capacity was related to fewer intrusions in the suppression condition. Furthermore, Rosen & Engle (1998) found that greater working memory capacity, as measured by OSPAN, was related to more successful suppression of intrusive thoughts. However, it should be noted that Waldhauser, Johansson, Bäckström and Mecklinger (2011) reported that suppression-induced forgetting on the TNT was not related to working memory capacity, indexed by the OSPAN. Nevertheless, taken together, the weight of evidence suggests that there is a relationship between working memory capacity and memory suppression.

With this in mind, it is notable that depression and dysphoria are associated with deficits in working memory capacity (Christopher & McDonalds, 2005; Hubbard et al., 2016, Joormann & Gotlib, 2008; Noreen & Ridout, 2010; Rose & Ebmeier 2006). Thus, it is plausible that impoverished working memory in depressed participants might play a significant role in their difficulties in intentionally forgetting.

Working memory is considered to be a system that uses controlled attention to maintain goal-relevant information in memory (Baddeley, 1996). It has been proposed that working memory capacity underpins the ability to use controlled sustained attention in the face of distraction, or when irrelevant information needs to be suppressed (Brewin & Beaton, 2002; Engle, Kane, & Tuholski, 1999; Rosen & Engle, 1998). According to Nyberg, Brocki, Tillman, & Bohlin, (2009) working memory exerts significant control over inhibitory processing. This position is supported by previous evidence (Roberts & Pennington, 1996; Engle & Kane, 2004; Miyake et al., 2000; Tsujimoto, Kuwajima, & Sawaguchi, 2007); thus, it would seem likely that individual differences in working memory capacity, via its contribution to inhibition, would contribute to the variability in intentional forgetting (Levy & Anderson, 2002, 2008). However, it should be noted that other researchers have questioned the relationship between working memory and inhibition. For example, Wilhelm, Hildebrandt and Oberauer (2013) reported that performance on tasks of working memory (including OSPAN) was not significantly related to inhibition (indexed using Simon and Flanker tasks). Similarly, Shao, Janse, Visser, and Visser, (2014) reported that performance on the OSPAN was not related to inhibition, measured using the Stop Signal paradigm. Nevertheless, in her review of the literature on executive functions, Diamond (2013) argued that “WM and inhibitory control support one another and rarely, if ever, is one needed but not the other” (pg. 143), but concluded that working memory and inhibition are independent functions that are related. This position was supported by Malagoli and Usai (2015) who conducted a latent variable analysis of performance on four measures of inhibition and three measures of working memory and confirmed that performance clustered on two separate factors, but that these factors were strongly related (*r* = 0.68). Taken together the evidence suggests that working memory is likely to be an important factor in understanding impaired suppression-induced forgetting in depression. This is important, as it could reveal working memory as a viable target for cognitive intervention to improve the ability of depressed individuals to prevent unwanted memories from coming to mind.

**Fig. 1:**
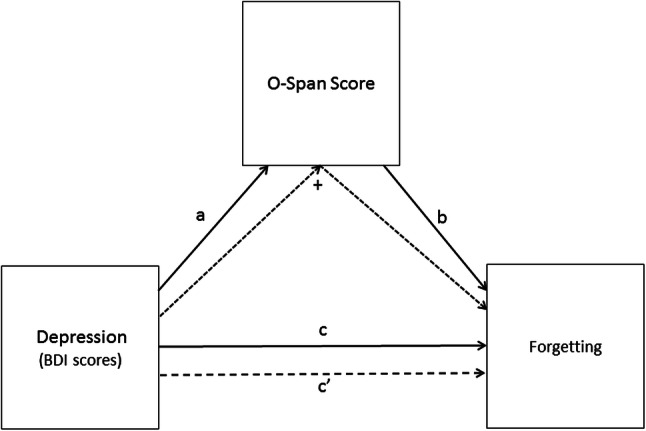
Illustration of the proposed mediation of the influence of depression on intentional forgetting by working memory capacity. a: the total effect of depression on working memory (OSPAN score), b: the direct effect of working memory on forgetting controlling for depression, c: the total effect of depression on forgetting, c′: the direct effect of depression on forgetting controlling for the influence of the mediator (OSPAN score) and +: the indirect effect of depression on forgetting via the mediator (OSPAN score)

Therefore, the aim of the present study was to determine if impaired suppression-induced forgetting in dysphoria is linked to variations in working memory. To this end, dysphoric and non-dysphoric participants were invited to learn a series of word pairs (neutral words paired with positive or negative adjectives). They were then presented with the cues from a subset of the pairs and asked to either recall the associated word (‘think’ condition) or to not recall (or think about) the target (‘no-think’ condition). To aid them in the ‘no-think’ condition, participants were provided with a substitute word for each cue and were asked to recall that word instead of the original target. Participants were subsequently given a final cued recall test, where they were asked to recall the targets to all cues (regardless of previous recall instructions). Prior to the TNT task, participants were invited to complete the operation Span task (OSPAN; Turner & Engle, 1989). In line with our previous findings (Noreen & Ridout, 2016a, b), we predicted that dysphoric participants would exhibit impaired suppression-induced forgetting of ‘to-be-forgotten’ words compared to non-dysphoric individuals. In line with Hubbard et al. (2016), we expected that dysphoric participants would exhibit poorer working memory capacity (lower OSPAN scores) than would the non-dysphoric group. We also predicted that forgetting would be negatively related to working memory capacity (scores on the OSPAN task). Finally, we expected that OSPAN score would mediate the effect of depression on intentional forgetting, such that there would be a significant indirect effect of depression on forgetting via working memory (see Fig. 1).

## Method

### Design

This study made use of a 2 × 2 × 2 × 3 mixed factorial design, with two between subjects factors; group (dysphoric and non-dysphoric) and word valence to be suppressed (positive and negative), and two within subjects factors: TNT instructions (respond and suppress) and repetitions (0, 2 and 8). The critical dependent variable was the percentage of words recalled on the final memory test. An additional dependent variable, the size of the suppression effect, was generated by calculating the difference in recall of words that were suppressed 2 and 8 times on the think–no think (TNT) task and words that were only presented during the initial learning phase of the TNT (baseline). Scores on the mood questionnaires (depression and anxiety) and the score on the working memory task (OSPAN) were included in analyses as continuous variables.

### Participants

One-hundred and sixty-eight students from Aston University were recruited for this study. At initial screening, participants completed a general health questionnaire developed by the experimenter, the Beck Depression Inventory-II (BDI-II; Beck, Steer, & Brown, 1996) and the trait scale of the State-Trait Anxiety Inventory (STAI-T, Spielberger, Gorsuch, Lushene, Vagg, & Jacobs, 1983). 11 participants were excluded as they had a history of a psychiatric illness, or had experienced a head injury, or were currently using medications that were deemed likely to have impaired cognitive functioning. Furthermore, an additional 17 participants were excluded based on their BDI-II scores.

Participants were allocated to groups based on their BDI-II scores. In line with previous research (Kao, Dritschel & Astell, 2006; Noreen & Ridout, 2016a, b), those that scored 5 or below were categorized at non-dysphoric and those that scored 15 and more were classified as dysphoric. This resulted in 60 dysphoric (23 M, 37 F; mean age = 21.83; SD = 4.9) and 61 non-dysphoric participants (20 M, 41 F; mean age = 22.67; SD = 5.4) taking part in the study, which took place 7–14 days after the initial screening.

### Tasks and measures

The *Beck Depression Inventory-II* (BDI-II; Beck et al., 1996) was used to assess depressed mood and allocate participants to dysphoric or non-dysphoric groups. BDI-II is a multiple choice, self-report inventory that looks at how an individual has been feeling in the preceding 2 weeks. BDI II consists of 21-items, with each item rated according to the severity of depression. Scores range from 0 to 63, with higher scores indicating more severe depression symptoms. This measure has shown to be valid and reliable (Arnau, Meagher, Norris, & Bramson, 2001).

The *State-Trait Anxiety Inventory* (STAI; Spielberger et al., 1983) was used to assess dispositional and situational anxiety, which is important as previous studies have reported a link between trait anxiety and impaired suppression induced forgetting (Benoit, Davies & Anderson, 2016; Kim, Yi, Yang & Lee, 2007; Marzi, Regina & Righi, 2014; Waldhauser et al., 2011). The STAI comprises two questionnaires, each containing 20 items that record the presence or absence of anxiety symptoms on a 4-point Likert scale. The latter are inverted for the purposes of calculating a total score. Scores range from 20 to 80 on each questionnaire with higher scores indicating more severe anxiety symptoms. Both subscales of this measure have been shown to be valid and reliable (Kabacoff, Segal, Hersen & Van Hasselt, 1997).

*The National Adult Reading Test* (NART; Nelson & Willison, 1991) is composed of a list of 50 words that are presented in order of increasing difficulty. The words are ‘irregular’ words that cannot be pronounced through the use of common phonetic rules. Participants are presented with one word at a time and are instructed to read the word out aloud. Responses are recorded so they can be scored. NART error score is the total number of reading errors made on the complete test. The number of NART errors has been shown to correlate negatively with full IQ score on the WAIS (Crawford et al., 1990), so it provides a proxy measure of intelligence. We used the NART to ensure variations in forgetting and working memory could not be ascribed to group differences in intelligence. Importantly, performance on the NART is unaffected by depression (Crawford, Besson, Parker, Sutherland, & Keen, 1987), which makes it ideal for estimating premorbid intelligence in dysphoric participants.

**Fig. 2:**
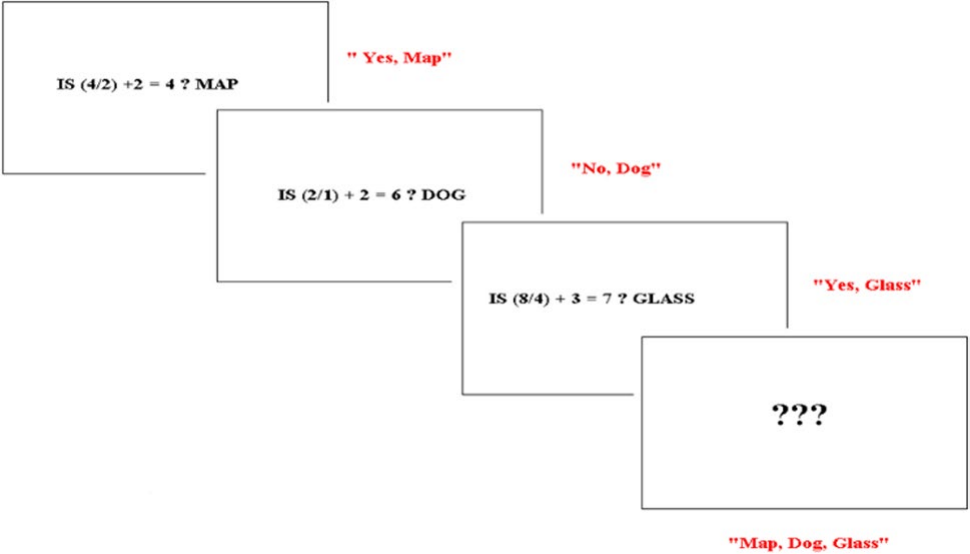
An example of one trial presented in the operation span with words task

*Operation span* (OSPAN; Unsworth, Heitz, Schrock, & Engle, 2005; Turner & Engle, 1989) was used to measure working memory capacity. OSPAN requires participants to solve a series of math operations while trying to remember a series of unrelated words. For each trial, participants are presented with a word and a simple arithmetic operation and asked to verify the answer to the operation and read the word aloud. Immediately after the participant reads the word, the next word and operation are presented. After the last operation string in each trial, participants are presented with a set of three question marks in the centre of the screen and are asked to write down the correct order of the words that followed the operation strings (see Fig. 2). One trial consists of a set of between two and five operation strings and words. After three practice trials, each containing two operation strings, participants receive the 12 experimental trials, three at each set size. The order of trials is arranged so that sets of different sizes are presented in a random order. Marks corresponding to the set size are allocated only if all the words in a trial are remembered in the correct order and if all arithmetic strings have been correctly verified. For example, if there were three operation strings in a trial and the three words were all recalled in the correct order, and all arithmetic strings were correctly verified, a score of 3 is given for that trial. A total score was calculated by adding up individual trial scores with scores ranging from 0 to 42.

*Think-No-think Task* (Anderson & Green, 2001): 72 adjective-noun pairs, drawn from Noreen & Ridout (2016a) were used in the current study as the experimental stimuli. These pairs consisted of 36 neutral nouns (e.g., person) paired with both a positive (e.g., happy) and a negative adjective (i.e., suffering). Each noun was also paired with a neutral adjective (e.g. tall), which were used as substitute words to aid suppression during the main TNT phase (see below). An additional ten word pairs, featuring different nouns paired with novel neutral adjectives, were created for use in the practice TNT phase and as filler items in the main TNT phase.

*Learning Phase* Participants were presented with the 36 nouns from the experimental set, half of which were paired with a positive adjective and half with a negative adjective. Word pairs were presented in six randomised blocks of six pairs. Each block also included one neutral-filler word pair (e.g., ‘underground cellar’) that remained the same across participants. Furthermore, two additional neutral word pairs were included at the beginning of the first block and two neutral word pairs were included at the end of the final block which remained the same for all participants. Each trial began with the presentation of a noun-adjective word pair on a computer screen for 6000 ms and participants were asked to create a self-referential image relating to the word pair and to rate the meaningfulness of this image on a scale of 1–5 (with 1 being not meaningful and 5 being very personally meaningful). Participants were given unlimited time to respond by pressing the key that corresponded to how meaningful the image was. This was then followed by a 600 ms inter-trial interval.

*Recall Phase* Participants were presented with a random sequence of the 46 nouns presented during the learning phase, each shown for 5200 ms, and were asked to recall aloud the associated target word (adjective) for each noun. They were then provided with feedback in the form of the correct target word being presented for 600 ms, followed by an inter-trial interval of 300 ms. Consistent with previous studies (Anderson & Green, 2001; Anderson et al., 2004), participants were required to achieve a minimum of 50% on the recall test to continue with the task. Participants were permitted three attempts to achieve this criterion, otherwise the experiment was terminated. All participants reached this criterion.

*Second learning phase* Before the main think/no-think phase, participants were presented with the 12 nouns that were about to be included in the suppression trials of the TNT. For half of the participants in each group (dysphoric/non-dysphoric), these cues had been paired with negative words during the learning phase; the remaining participants had learnt positive associates to these cues. These cues were now paired with a novel neutral word. Word pairs were presented one at a time (for 3000 ms) in a randomized order and participants were instructed to learn the new pairings, but to never think about the original target word that had been associated with each cue.

*Think/No*-*Think Training Phase* Prior to the main think–no think phase, participants completed random sequence of 26 practice trials, where the cues from nine of the filler word pairs were presented in green ink (each of which were presented twice in the sequence) and the cue from the remaining filler word pair was presented eight times in sequence, always in red ink. Participants were asked to recall the associated target for cues presented in green ink and to suppress (not think about) the target for the cue presented in red. The timings of these trials were identical to the main TNT trials (see below).

*Think/No*-*Think Phase* Participants were presented with a random sequence of 184 experimental trials. There were 124 ‘think’ trials, 60 of which consisted of the cues from 12 experimental word pairs presented in green ink (six cues were presented twice in the sequence and six were repeated eight times). Half of the participants had learned positive associates to these cues during encoding and half had learned negative associates. The remaining 64 ‘think’ trials featured the cues from eight filler word pairs (each repeated eight times in green ink). There were also 60 ‘no-think’ trials, which consisted of the nouns from 12 of the experimental word pairs presented in red ink (six were presented twice in the sequence and six were repeated eight times). During the learning phase, participants who had learned positive associates to cues presented on the ‘think’ trials learned negative associates to the ‘no-think’ cues and vice versa. Each trial began with a small cross appearing on the screen for 200 ms, followed by a cue word for 3000 ms. On ‘think’ trials (green ink), participants were asked to recall the associated target word. Incorrect responses on ‘think’ trials resulted in the correct target being displayed for 500 ms in blue. On ‘no-think’ trials (red ink), participants were told not to think about the associated target word. To help them do this, they were asked to try and recall the neutral substitute words that had been paired with these cues during the second learning phase. ‘No-think’ trials were preceded by 3 very large red Xs (displayed for 500 ms) as a cue for suppression. This was in line with previous research, which has found stronger forgetting effects when suppression trials are primed (Hanslmayr, Leipold & Bauml, 2010; Noreen and Ridout, 2016a, b). Following ‘no-think’ trials, the substitute word was presented for 500 ms. Trials (in both ‘think’ and ‘no-think’ conditions) were separated by an inter-trial interval of 400 ms.

*Final Recall Test Phase* Participants were presented with all thirty six cues from the experimental trials and were asked to recall the original target words, regardless of previous recall instructions. Each trial began with a cross being displayed in the centre of the screen (for 200 ms) followed by the cue word (for 4000 ms) and participants were asked to recall aloud the associated target word for the cue. Participants were told that if more than one word came to mind they should report both, but everyone was reminded that it was very important to try and recall the original target word. Trials were separated by a 400 ms inter-trial interval.

### Procedure

During the initial screening, participants completed a general health questionnaire, the BDI, and the trait scale of the STAI. During the main experimental session, all participants completed the tasks and measures in the following order: OSPAN task, TNT task, NART, BDI and the state scale of the STAI.

## Results

### Participant characteristics

Participants’ age, NART error scores, OSPAN, BDI and STAI scores (see Table 1) were analysed using separate independent *t* tests. Our analyses revealed that dysphoric and non-dysphoric participants did not differ in age or in their general intellectual ability (i.e. NART scores); all tests *p* > 0.05. Contrary to expectations, we found that there was no significant group difference on the OSPAN, which suggests that overall both groups were matched in terms of their working memory capacity; *t*(119) = 0.15, *p* = 0.88. As expected, dysphoric individuals scored significantly higher on state and trait anxiety than did the non-dysphoric participants; *t*(119) = 6.65, *p* < 0.001 and *t*(119) = 5.82, *p* < 0.001, respectively. This group difference in anxiety was controlled for in subsequent analyses.

**Table 1:** Mean indices for age, National Adult Reading Test (NART) errors, mood measures, and working memory (operation span) as a function of participant group (standard deviations are presented in parentheses)

	Dysphoric (*n* = 60)	Non-dysphoric (*n* = 61)	*p* value
Age (years)	21.83 (4.93)	22.67 (5.36)	ns
NART	17.0 (7.24)	17.41 (7.39)	ns
BDI-II	19.18 (4.89)	3.06 (1.66)	< 0.001
STAI-S	42.37 (10.66)	31.21 (7.56)	< 0.001
STAI-T	45.0 (11.11)	34.62 (8.34)	< 0.001
OSPAN	25.02 (10.45)	25.30 (10.07)	ns

*NART* National Adult Reading Test error score, *BDI-II* Beck Depression Inventory, *STAI-S* State-Trait Anxiety Inventory—state subscale, *STAI-T* State-Trait Anxiety Inventory—trait subscale, *OSPAN* Operation Span score

### Memory accuracy

**Fig. 3:**
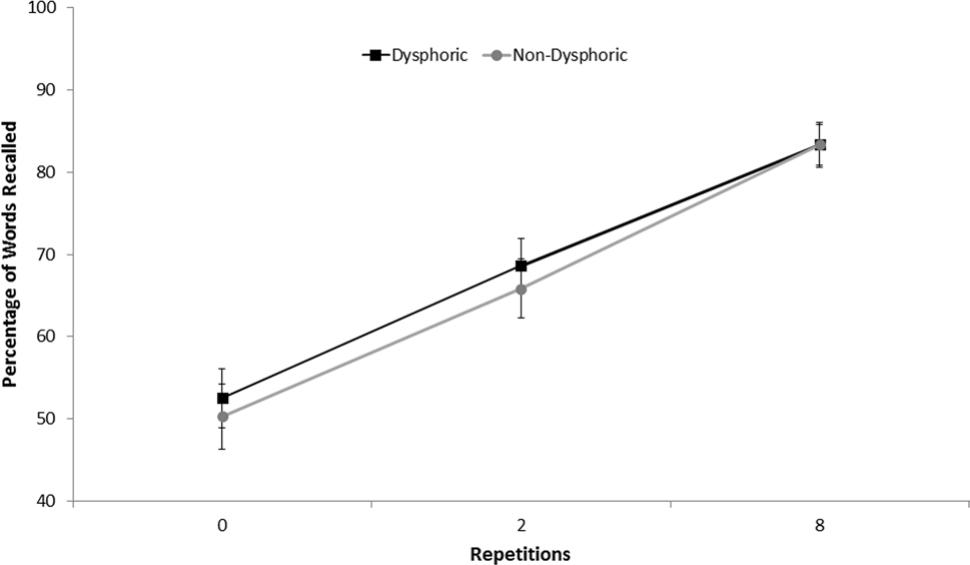
Mean percentage of ‘think’ words correctly recalled by the dysphoric and non-dysphoric groups (error bars represent + one standard error of the mean)

The percentage of words correctly recalled on the final memory test were analysed using a 2 (group; dysphoric vs. non-dysphoric) × 2 (valence for suppression; positive vs. negative) × 2 (instruction; ‘think’ vs. aided ‘no-think’) × 3 (repetition; 0 vs. 2 vs. 8) mixed factorial ANOVA. Our analysis revealed main effects of group, *F* (1, 119) = 12.85 *p* < 0.001, *η*_*p*_^2^ = 0.10, instruction, *F* (1, 117) = 76.5 *p* < 0.001, *η*_*p*_^2^ = 0.40, and repetition, *F* (2, 118) = 26.94 *p* < 0.001, *η*_*p*_^2^ = 0.19. However, these need to be considered in light of a significant group × instruction × repetition interaction, *F* (2, 118) = 7.20, *p* = 0.001, *η*_*p*_^2^ = 0.06. Subsequent analyses revealed that, in the ‘think’ condition (see Fig. 3), dysphoric participants recalled significantly more words presented twice (*M* = 68.61, SD = 25.69) and eight times (*M* = 83.33, SD = 21.26); than baseline words (*M* = 52.50, SD = 28.09); *t*(59) = 4.55, *p* < 0.001 and *t*(59) = 7.71, *p* < 0.001, respectively. Similarly, non-dysphoric participants recalled a greater number of the words presented twice (*M* = 65.68, SD = 28.29) and eight times (*M* = 83.33, SD = 19.48) in comparison to baseline (*M* = 50.27, SD = 30.50); *t*(60) = 2.94, *p* < 0.01 and *t*(60) = 8.15, *p* < 0.001, respectively. Importantly, the dysphoric and non-dysphoric groups did not differ in their recall of these words.

**Fig. 4:**
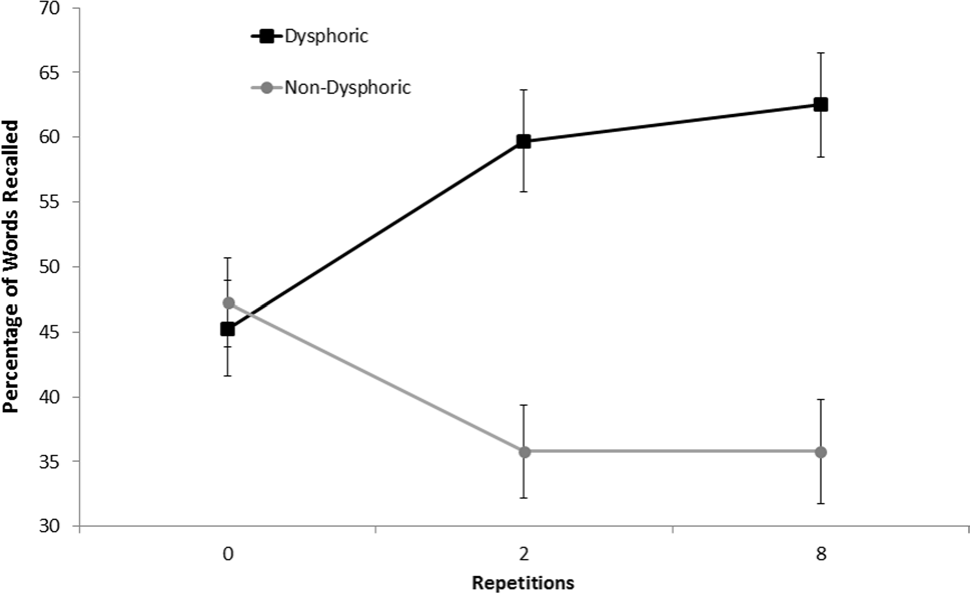
Mean percentage of ‘no-think’ words correctly recalled by the dysphoric and non-dysphoric groups (error bars represent ± one standard error of the mean)

In the ‘no-think’ condition (see Fig. 4), the dysphoric group recalled significantly more words presented twice (*M* = 59.72, SD = 30.73) and eight times (*M* = 62.50, SD = 31.39) than baseline words (*M* = 45.28, SD = 28.47); *t*(59) = 2.88, *p* < 0.01 and *t*(59) = 3.02, *p* < 0.01, respectively. On the other hand, the non-dysphoric group recalled significantly fewer words presented twice (*M* = 35.79, SD = 28.03) and eight times (*M* = 35.79, SD = 31.30) than baseline words (*M* = 47.27, SD = 26.56); *t*(60) = 2.42, *p* = 0.019 and *t*(60) = 2.11, *p* = 0.04. Taken together, these findings suggest that only the non-dysphoric group were successful at demonstrating suppression-induced forgetting.^1^

Our analysis also revealed a significant group × valence interaction, *F* (1, 117) = 8.49, *p* < 0.01, *η*_*p*_^2^ = 0.07, with subsequent analyses demonstrating that the dysphoric group recalled significantly more negative (*M* = 66.76, SD = 12.94) than positive words (*M* = 57.22, SD = 16.19), regardless of recall instructions; *t*(58) = 2.52, *p* = 0.014. However, the non-dysphoric group showed no significant differences in their recall of positive (*M* = 55.56, SD = 14.28) and negative words (*M* = 50.63, SD = 10.70); *t*(59) = 1.53, *p* = 0.13.

### Memory accuracy for substitute words

To explore whether there were differences in the recall accuracy of substitute words presented in the ‘no-think’ condition, we conducted a 2 (group; dysphoric vs. non-dysphoric) × 2 (valence for suppression; positive vs. negative) × 3 (repetition; 0 vs. 2 vs. 8) mixed factorial ANOVA. This analysis revealed a main effect of repetition, F1, 117) = 26.13, *p* < 0.001, with participants recalling more aided words presented eight times (*M* = 57.44, SD = 26.96) than twice (*M* = 39.39, SD = 25.91). These findings are consistent with those obtained by previous research which has found recall of substitutes on average of 34% for words presented two times and 56% for words presented 12 times (Hertel & Calcaterra, 2005). This analysis revealed no other significant main effects or interactions; all tests *F* < 1.

### Relationship between working memory capacity and forgetting

We calculated the size of the suppression-induced forgetting effect by subtracting ‘no-think’ scores for words repeated two and eight times from baseline scores, with negative scores indicating successful forgetting. Overall, the size of the suppression-induced forgetting effect for both two and eight repetitions was significantly negatively related to working memory (OSPAN score); *r*(121) = − 0.23, *p* < 0.05 and *r*(121) = − 0.24, *p* < 0.01, respectively.

### Relationship between depression and working memory capacity

We conducted separate regression analyses for dysphoric and non-dysphoric participants to determine if depression scores predicted working memory (OSPAN scores). For the dysphoric group, we found that depression accounted for 7% of the variance in working memory capacity; *R*^2^ = 0.07, *R*^2^ adjusted = 0.06, *F* (1, 58) = 4.51, *p* = 0.04, *β* = 0.27, SE = 0.27, *p* = 0.04.^2^ On the other hand, depression scores did not significantly predict working memory capacity in the non-dysphoric group; *F* (1, 59) = 0.03, *p* = 0.87.

### Mediation analysis

**Fig. 5:**
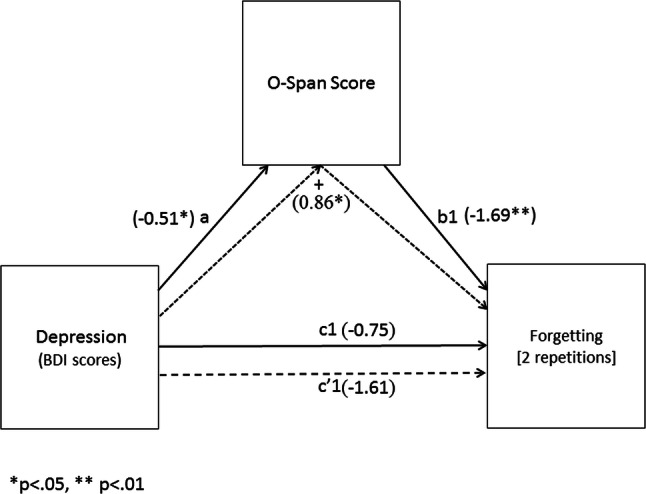
Mediation model for the direct and indirect effects of depression on forgetting [2 repetitions]

**Fig. 6:**
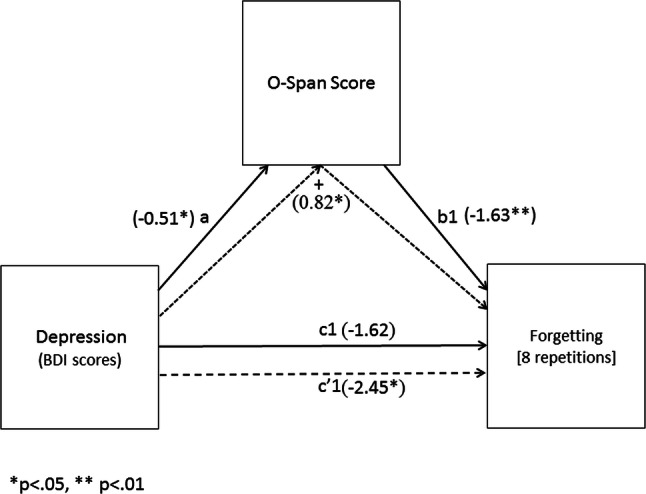
Mediation model for the direct and indirect effects of depression on forgetting [8 repetitions]

To test the prediction that the effect of depression on forgetting would be mediated by working memory capacity, we used a bootstrapping procedure on the dysphoric participants’ data to compute the 95% CI around the indirect effect (i.e., the path through the mediator) using the PROCESS macro in SPSS (Model 4; Hayes, 2013). The paths for these models can be derived from Figs. 5 and 6 and their corresponding coefficients and 95% CIs from Table 2. In each mediation analysis, depression was entered as the independent variable with working memory capacity entered as the mediator. The first model examined mediation for forgetting of words presented twice and the second model examined mediation for forgetting of words presented eight times.

**Table 2:** Path coefficients and confidence intervals from the mediation analyses estimated using PROCESS

Path estimates	Coefficient (SE)	LLCI	ULCI
a	− 0.51 (0.24)*	− 0.99	− 0.02
b1	− 1.69 (0.46)**	− 2.61	− 0.78
b2	− 1.63 (0.53)**	− 2.69	− 0.57
c1	− 0.75 (0.93)	− 2.61	1.10
c2	− 1.62 (1.04)	− 3.71	0.47
c′1	− 1.61 (0.87)	− 3.35	0.14
c′2	− 2.45 (1.01)*	− 4.47	− 0.42

Indirect effects	Effect (SE)	LLCI	ULCI
Model 1	0.86 (0.40)^a^	0.17	1.75
Model 2	0.82 (0.42)^a^	0.18	1.89

*LLCI* 95% lower-limit confidence interval, *ULCI* 95% upper-limit confidence interval, *a* path from depression to working memory, *b1* path from working memory to forgetting [2 repetitions], *b2* path from working memory to forgetting [8 repetitions], *c1* path from depression to forgetting 2 in the model without the mediator, *c2* path from depression to forgetting 8 in the model without the mediator, *c′1* direct effect of depression on forgetting 2 in the model with working memory included, *c′2* direct effect of depression on forgetting [8 repetitions] in the model with working memory included, *Model 1* forgetting [2 repetitions]; Model 2 = forgetting [8 repetitions]

*p < 0.05; **p < 0.01

^a^Indirect effect

In the first model (see Fig. 5), the path from depression to working memory capacity (a) was significant, as was the path from working memory capacity to forgetting (b1). However, neither the path from depression to forgetting (c1) nor the path from depression to forgetting when controlling for working memory capacity (c′1) was significant. Critically, the indirect effect (+) of depression on forgetting via working memory capacity was significant, confirming that working memory capacity mediated the effect of depression on forgetting [2 repetitions].

In the second model (see Fig. 6), the path from depression to working memory (a) and the path from working memory to forgetting (b2) were both significant. The path from depression to forgetting 8 (c2) was not significant, although the path from depression to forgetting, when controlling for working memory capacity (c′2), was significant. Importantly, the indirect path from depression to forgetting via working memory (+) was significant, showing that working memory capacity mediated the effect of depression on forgetting [8 repetitions].^3^

## Discussion

The aim of the present study was to determine if variations in working memory function would explain impaired suppression-induced forgetting in subclinical depression (dysphoria). Dysphoric and non-dysphoric participants learned a series of emotional cue–target word pairs, before being presented with a subset of the cues and asked to either recall the associated target (think) or to prevent it from coming to mind (no think). Participants were subsequently asked to recall the targets to all cues (regardless of previous recall instructions). As expected, we found that the non-dysphoric participants demonstrated successful suppression-induced forgetting, as, for both the two and eight repetition conditions, they recalled significantly fewer ‘no think’ words in comparison to baseline. These findings are consistent with previous studies demonstrating successful forgetting in healthy participants (Noreen & Ridout, 2016a, b; Hertel & Calcaterra, 2005; Joormann et al., 2009). Interestingly, our study did not show increased suppression-induced forgetting between the two and eight repetition conditions. Although this is inconsistent with some previous findings (Anderson & Green, 2001; Anderson et al., 2004; Noreen & MacLeod, 2013, 2014, 2015; Noreen, Bierman & MacLeod, 2014), it should be noted that other studies have also reported no significant increase in the size of the suppression-induced forgetting effect with increased repetitions (Hertel & Calcaterra, 2005). One reason for this discrepancy may relate to the fact that different studies have used a different number of repetitions. For example, Anderson & Green (2001) used one, eight and sixteen repetitions whilst Hertel & Calcattera (2005) used 2 and 12 repetitions. As we used two and eight repetitions, which more closely resemble the study of Hertel & Calcaterra (2005), it is perhaps not surprising that we did not find greater suppression-induced forgetting with increased repetitions.

In line with our predictions, we found that dysphoric participants were unsuccessful at forgetting, even with the aid of a thought substitution strategy and actually showed enhanced recall of the ‘no-think’ words relative to baseline words. These findings are consistent with our previous work (Noreen & Ridout; 2016a, b) and confirm that thought substitution may not be an effective strategy to help depressed individuals intentionally forget unwanted memories, which is inconsistent with the findings of other studies (e.g. Joormann et al. (2009). One possible explanation for the discrepancy in findings is the nature of the cues and substitutes that were used in the different studies. For example, in the current study we used related and meaningful cue–target word pairs (in line with Noreen & Ridout, 2016a, b); in contrast, Joormann et al. (2009) used unrelated cue–target word pairs, which may have been more difficult to learn and easier to suppress (Hertel & Mahan, 2008). Furthermore, Joormann et al. (2009) used substitutes that were more closely related to the cues (e.g., mushroom-poison) than were the original targets (e.g., mushroom-hostage), thus making them easier to recall. Using unrelated word pairs at encoding and highly related substitutes during suppression may have exaggerated the effectiveness of the thought substitution strategy in suppressing memories in depression. Alternatively, it is also possible that the self-referential encoding of the word pairs in our study may have led to greater integration between cues and the original response items, which may have made it more difficult for depressed participants to intentionally forget the unwanted items.

The prediction that dysphoric participants would exhibit poorer working memory (lower OSPAN scores) in comparison to the non-dysphoric group was not supported by our data. This is not consistent with the findings of previous studies (Christopher & McDonalds, 2005; Hubbard et al., 2016, Joormann & Gotlib, 2008; Noreen & Ridout, 2010; Rose & Ebmeier 2006). One possible explanation concerns the participant sample used in the different studies. Previous research has often involved clinically depressed patients whereas in the current study we examined working memory in participants with subclinical depression. As dysphoric samples may represent a milder form of depressed mood, it is possible that our dysphoric sample may not have exhibited depressive symptoms severe enough to impair working memory. This is supported by our analyses which showed that within the dysphoric participants at least, greater depression severity (i.e., higher BDI scores) was associated with poorer working memory function. It is also important to mention that our dysphoric and non-dysphoric sample consisted of university students with equivalent intellectual capacity, which may have masked overall group differences; this is supported by the finding that the two groups were matched on their NART performance.

As predicted, suppression-induced forgetting in both the two and eight repetition conditions was significantly related to working memory. Furthermore, an important and novel finding of our study was that working memory mediated the effect of depression on forgetting in both the two and eight repetition conditions. These findings are consistent with previous research demonstrating an important role for working memory in successful intentional forgetting (Aslan and Bäuml, 2011; Noreen & De Fockert, 2017), but also represent an important development to the research on forgetting in depressed states. However, the question remains as to whether it is the capacity element or the executive control aspect of working memory that is underlying these findings.

Given that intentional forgetting on the TNT involves preventing unwanted information from coming to mind, then it would seem likely that the executive control aspect of working memory would be the key factor, as this enables goal-relevant information to be kept in mind temporarily, whilst preventing irrelevant information from coming to mind (Goldman-Rakic, 1996; Cowan, 2001; Baddeley, 2012). Consistent with this notion is the large body of research which suggests that suppression-induced forgetting effects are due to an inhibitory mechanism that disrupts the availability of the unwanted memory which later renders in inaccessible (Anderson & Hansmayr, 2014; Anderson et al., 2004). This is also supported by evidence of right dorsolateral prefrontal cortex (DLPFC) activation during memory suppression (Anderson et al., 2004; Depue et al., 2007; Benoit & Anderson, 2012; Gagnepain, Henson & Anderson, 2014; Benoit, Hulbert, Huddleston, & Anderson, 2015; Depue et al. 2015), which has been interpreted as active inhibition of the unwanted memories from entering conscious awareness (Anderson et al., 2004). However, it is important to mention that although the OSPAN is considered to measure both aspects of working memory, capacity and attentional control (Arnell, Stokes, MacLean & Gicante, 2008), previous findings have reported weak relationships between OSPAN and measures of inhibition. For example, Wilhelm et al. (2013) reported no relationship between performance on the OSPAN and measures of inhibition (indexed using Simon and Flanker tasks). Similarly, Shao et al. (2014) reported that scores on the OSPAN were not related to performance on the Stop Signal paradigm. However, as noted by Diamond (2013) there are different forms of inhibition, which may be served by different neural substrates. The tasks reported above involve behavioural inhibition, which is different from the form of inhibition required for the TNT, i.e. resisting unwanted thoughts and memories. Diamond termed the latter ‘cognitive inhibition’ and argued that it is more closely related to working memory than inhibitory control. With this in mind, it would seem likely that the current findings would be better explained by the executive control aspect of working memory rather than WM capacity. Further work is required to confirm this using ‘purer’ working memory tasks, such as the digit span (forward and backwards), that are better able to isolate to two aspects of working memory.

It should be noted that the pattern of mediation in the current study was slightly different for the two and eight repetition conditions. In the two repetition condition, the direct effect of depression (controlling for working memory capacity) was non-significant, but the indirect effect of depression via working memory was significant, which suggests that depression only influenced forgetting in this condition via its effect on working memory, perhaps by reducing available working memory resources that could be used to control the suppression of the words. On the other hand, in the eight repetition condition depression exerted both a direct effect on forgetting (after controlling for working memory capacity) and an indirect effect via working memory. This again suggests that depression influenced forgetting via its effect on working memory. However, depression also exerted an independent effect on forgetting, with greater depression severity associated with poorer forgetting. This could simply be a consequence of negative mood, as our previous work has shown that healthy participants induced into a negative mood exhibited impaired suppression-induced forgetting (Noreen & Ridout, 2016b). However, this does not adequately explain why negative mood had a direct impact on forgetting in the eight repetition condition but not the two repetition condition. It is plausible that increasing number of attempts to ‘not think’ about the target information may lead to a greater number of intrusions of the unwanted memory, putting greater demands on the executive control processes (Noreen & Ridout 2016b). Consistent with this view, previous studies have reported that depression is associated with difficulties engaging in effortful processing (Beevers, 2005; Hartlage, Alloy, Vazquez, & Dykman, 1993). One explanation for this is that depressed mood acts as an additional cognitive load—akin to performing a dual task (e.g. Beevers, 2005). Evidence for this comes from Bredemeier et al. (2012) who demonstrated that depression resulted in equivalent deficits on a selective attention task to those observed in non-depressed participants completing a dual task. Like dual-task studies, under conditions of low cognitive demand depression has no direct effect on the performance of a primary task, but as the demands of the primary task increase depressed mood begins to exert its effect, perhaps due to the allocation of resources to processing of task irrelevant material in the environment (Jones, Siegle, Muelly, Haggerty, & Ghinassi, 2010) or task-irrelevant internal processing such as rumination (Beevers, 2005; Levens et al., 2009). The finding that working memory mediated this influence of depression on forgetting in both two and eight repetition conditions is important, as it suggests that cognitive training to improve working memory could potentially increase the ability of individuals with depression to prevent unwanted memories from coming to mind, which in turn could have positive effects on emotion regulation and ongoing mood. This is consistent with a recent proposal by Engen and Anderson (2018) who suggested that memory control is fundamental to emotion regulation, with other factors, such as working memory capacity and executive control abilities also influencing this relationship. With this in mind, it is notable that there is a growing body of evidence that working memory training can improve inhibitory control. For example, in a series of studies, Klingberg and colleagues demonstrated that working memory training over a 5-week period significantly improved working memory and inhibitory control in children (Klingberg, Forssberg & Westerberg, 2002; Klingberg et al., 2005). Furthermore, Borella, Carretti, Riboldu & De Beni (2010) demonstrated that working memory training led to improvements in inhibition in older adults (65–75 years). Notably, Aasvik et al. (2017) reported that WM training improved inhibition in a group of participants who were on sick leave due to complex issues (including depression and anxiety). These findings are consistent with neuroimaging evidence that working memory and inhibitory tasks both activate the ventrolateral prefrontal cortex, which might reflect the neural basis for transfer between working memory and inhibition (McNab et al., 2008). However, these findings need to be considered with caution, as other studies have not found that working memory training improves inhibitory control (Melby-Lervag & Hulme, 2013; Schwaighofer, Fischer & Bühner, 2015; Shipstead, Redick & Engle, 2012; also see Diamond & Lee, 2011). Nevertheless, there is arguably sufficient evidence to warrant further research to determine if working memory training would improve forgetting in participants with depression. An additional finding of the current study was that dysphoric participants showed enhanced memory for negative relative to positive words, irrespective of condition (‘think’ ‘no-think’ or baseline). This memory bias for negative words is consistent with the findings of previous research (Watkins, Mathews, Williamson, & Fuller, 1992; Noreen & Ridout, 2016a, b).

One limitation of the current study is that we did not take a subjective measure of how well participants complied with instructions during the TNT phase. Previous studies have included a compliance questionnaire after the task in order to ascertain how well participants had complied with ‘think’ and ‘no-think’ instructions and have reported that forgetting was related to compliance (Anderson & Green, 2001; Hertel & Calcaterra, 2005; Noreen & MacLeod, 2013, 2014; Noreen & Ridout, 2016a, b). It is possible that dysphoric participants, and those with low working memory capacity, may have been less compliant at the following ‘no think’ instructions, which could explain the deficit in intentional forgetting. However, it is important to note that in our previous study (Noreen & Ridout, 2016a) depression was only associated with non-compliance in the unaided condition; thus, as we used aided suppression in the current study it is less likely that the current findings were due to problems in compliance. Nonetheless, future research should include a compliance questionnaire to determine if suppression-induced forgetting effects related to depression and/or working memory capacity are due to noncompliance with ‘no think’ instructions.

Another issue to consider is the group difference in state and trait anxiety. It is plausible that anxiety and not depression could have been influencing working memory and forgetting. However, when all data were reanalysed controlling for anxiety all the key findings remained, which strongly suggests that it was depression and not anxiety that was impairing working memory and forgetting. The current findings are inconsistent with the previous research reporting a link between trait anxiety and memory suppression (Benoit, Davies & Anderson, 2016; Kim et al., 2007; Marzi, Regina & Righi, 2014; Waldhauser et al., 2011). One possible explanation for the discrepancy in findings is the valence of the materials used across studies. Our study used word pairs that were essentially depression relevant in nature, whilst previous studies used more generic negative material, which may reflect more threat-relevant information. Given that previous research has found that anxious individuals demonstrate specific memory biases for threat-relevant information (Herrera, Montorio, Cabrera & Botella, 2017), it is possible that the word pairs used in the present study were not threatening enough to elicit such biases and, thus, may not have led to impaired suppression-induced forgetting. Another issue of note is that none of the studies, cited above, reporting an influence of trait anxiety on forgetting had controlled for the presence of depression in their samples. Given that trait anxiety and depression are highly correlated in student and clinical samples (Clark & Watson, 1991; Mook, Van Der Ploeg, & Kleijn, 1990; Watson, 2009; Raes, 2010) it is possible that uncontrolled depression might have influenced forgetting in these previous studies.

In conclusion, dysphoric participants demonstrated the expected deficit in suppression-induced forgetting relative to their non-dysphoric counterparts and instead showed enhanced memory for to-be-forgotten material. Also as expected, working memory function was negatively related to forgetting. Furthermore, as expected, working memory mediated the effect of depression on forgetting in both the two and eight repetition conditions. This represents an important novel contribution to the understanding of impaired suppression-induced forgetting in depression and highlights the potential of working memory training as an intervention for improving control of memory (and possibly mood) in depressed individuals.

## Footnotes

1 The data were reanalysed using an ANCOVA, with state and trait anxiety entered as covariates, and the results were unchanged, we therefore report the results of the initial analysis.

2 The data were reanalysed using a hierarchical regression with state and trait anxiety entered at the first step and depression at the second step. Anxiety did not predict working memory performance and the observed relationship between depression and working memory was unaffected, we therefore report the initial analysis.

3 Both mediation models were recalculated with state and trait anxiety entered as covariates. The results of both mediation models were unchanged, we therefore report the initial analysis.
